# Co-culture model of B-cell acute lymphoblastic leukemia recapitulates a transcription signature of chemotherapy-refractory minimal residual disease

**DOI:** 10.1038/s41598-021-95039-x

**Published:** 2021-08-04

**Authors:** Stephanie L. Rellick, Gangqing Hu, Debra Piktel, Karen H. Martin, Werner J. Geldenhuys, Rajesh R. Nair, Laura F. Gibson

**Affiliations:** 1 Department of Microbiology, Immunology and Cell Biology, West Virginia University School of Medicine, 1 Medical Center Drive, Morgantown, WV 26506 USA; 2 Department of Pharmaceutical Sciences, West Virginia University School of Pharmacy, Morgantown, WV 26506 USA; 3 West Virginia University Cancer Institute, Morgantown, WV 26506 USA; 4 Bioinformatics Core, West Virginia University, Morgantown, WV 26506 USA; 5 West Virginia Clinical and Translational Science Institute, Morgantown, WV 26506 USA

**Keywords:** Cancer microenvironment, Haematological cancer, Cancer, Computational biology and bioinformatics

## Abstract

B-cell acute lymphoblastic leukemia (ALL) is characterized by accumulation of immature hematopoietic cells in the bone marrow, a well-established sanctuary site for leukemic cell survival during treatment. While standard of care treatment results in remission in most patients, a small population of patients will relapse, due to the presence of minimal residual disease (MRD) consisting of dormant, chemotherapy-resistant tumor cells. To interrogate this clinically relevant population of treatment refractory cells, we developed an in vitro cell model in which human ALL cells are grown in co-culture with human derived bone marrow stromal cells or osteoblasts. Within this co-culture, tumor cells are found in suspension, lightly attached to the top of the adherent cells, or buried under the adherent cells in a population that is phase dim (PD) by light microscopy. PD cells are dormant and chemotherapy-resistant, consistent with the population of cells that underlies MRD. In the current study, we characterized the transcriptional signature of PD cells by RNA-Seq, and these data were compared to a published expression data set derived from human MRD B-cell ALL patients. Our comparative analyses revealed that the PD cell population is markedly similar to the MRD expression patterns from the primary cells isolated from patients. We further identified genes and key signaling pathways that are common between the PD tumor cells from co-culture and patient derived MRD cells as potential therapeutic targets for future studies.

## Introduction

B cell acute lymphoblastic leukemia (ALL) develops when immature B cells stop differentiating and begin rapidly proliferating, allowing for an accumulation of immature cells in the bone marrow as well as the periphery^1^. While most ALL patients achieve remission following the standard-of-care treatment regimen, there is a subset of patients that relapse with poor prognostic outcomes^2,3^. Relapsed disease is often due to an expansion of minimal residual disease (MRD) that remains in the bone marrow following treatment, as the marrow is a unique protective site^4^. The presence of MRD following completion of treatment is one of the most important prognostic indicators for refractory or relapsed disease^5,6^. The cells comprising this MRD population are often dormant and chemotherapy-resistant, making the current standard-of-care therapies ineffective in achieving eradication of disease^7–9^. Therefore, identifying new therapies that specifically target the MRD cells is critical^7,8^.

**Figure 1:**
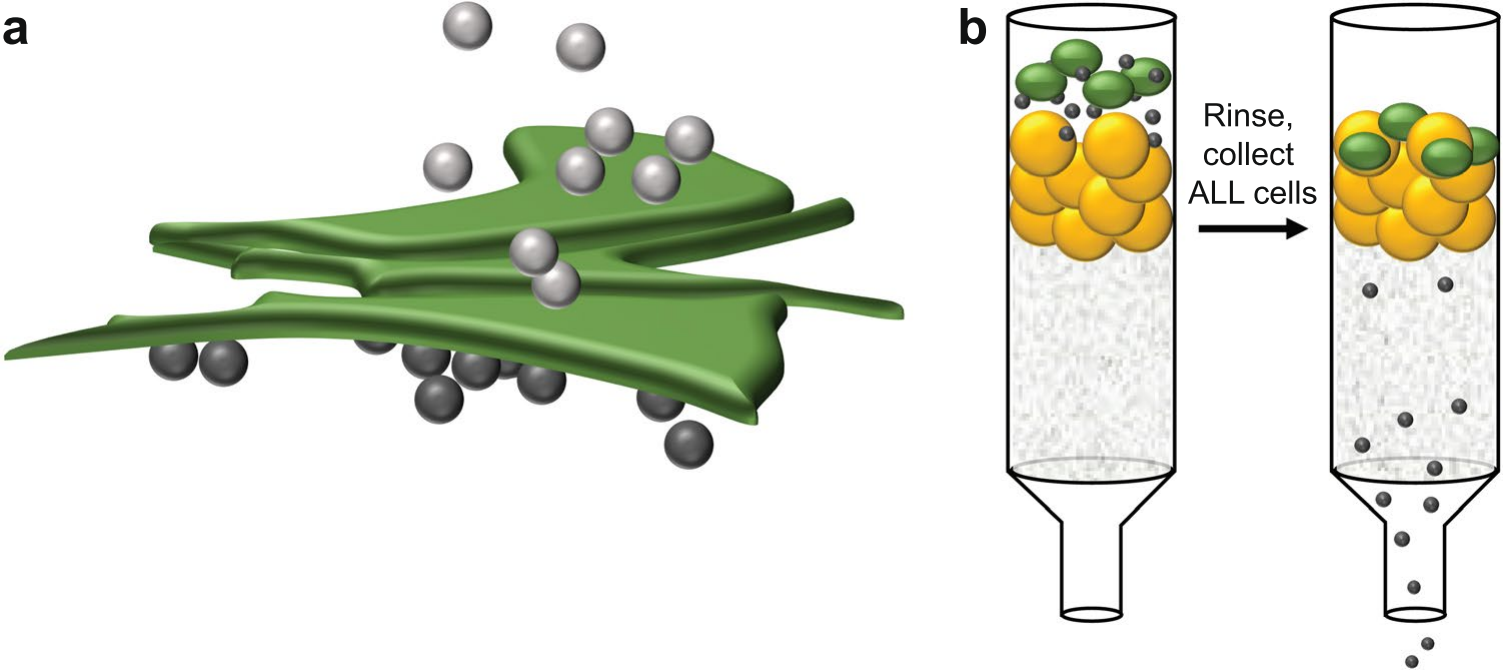
Schematic of in vitro co-culture model. (**a**) A representation of the three cell populations in the co-culture with the suspension and phase bright cells (light grey circles), phase dim cells (PD-dark grey circles), and BMSC or HOB (adherent cells-green). (**b**) To isolate the PD cells, after removing the media and rinsing to remove cells attached to the top of the adherent cells, the adherent cells and PD cells are collected by trypsinization and purified by size exclusion using Sephadex G-10 (yellow circles).

There is limited literature available in which MRD has been phenotypically characterized, and most studies were aimed at detection of MRD to improve outcome^9,10^. To evaluate potential therapeutics for chemotherapy-resistant ALL, an in vitro co-culture model system was developed by our laboratory that provides a more clinically relevant cell population compared to growing leukemic cells in media alone^11–14^. In this model, B-cell ALL cells are grown in co-culture with either bone marrow stromal cells (BMSC) or human osteoblasts (HOB), which are two of the cellular components in the bone marrow microenvironment that are critical for support of hematopoiesis^7,11–13^. BMSC and HOB niches within the bone marrow also provide a protective environment for leukemic cells, and interaction with these components conveys chemotherapy resistance and the induction of quiescence^15^. ALL cells in this co-culture system segregate into three sub-populations, with tumor cells floating in suspension above the adherent cells (S), cells in contact with the top of the adherent layer that appear bright in phase contrast microscopy (PB), and those that bury underneath the adherent cell layer that appear phase dim (PD) (Fig. 1). Sub-populations can be isolated, and previous functional studies have shown that the PD cells are more resistant to chemotherapeutic agents, have a reduction in proliferation, with a greater number of cells in G0/G1, and metabolism is more glycolytic when compared to cells grown in media alone^16^. Growing B-cell ALL in this co-culture provides a clinically relevant in vitro model to study refractory disease, and allows for the evaluation of potential therapeutic strategies that include the influence of the bone marrow microenvironment.

In the current study, we utilized RNA-Seq to define the expression patterns in the different sub-populations of the co-culture, including the PB ALL cells, the PD ALL cells (MRD, or refractory disease surrogate population), and the ALL cells grown in media alone (long term media culture, LTMC). Through an integrative analysis with a previously published expression data set that defined transcription signatures for MRD cells from relapsed patients^17^, we found that the co-culture PD cells exhibited an expression pattern similar to that of patient MRD cells. These results demonstrate that our in vitro co-culture model is biologically more relevant to MRD than the tumor cells cultured in media alone. As such, it can serve as one tool in developing and testing new therapeutics, prior to evaluation in vivo, to specifically target cells that contribute to relapse of ALL.

## Results

**Figure 2:**
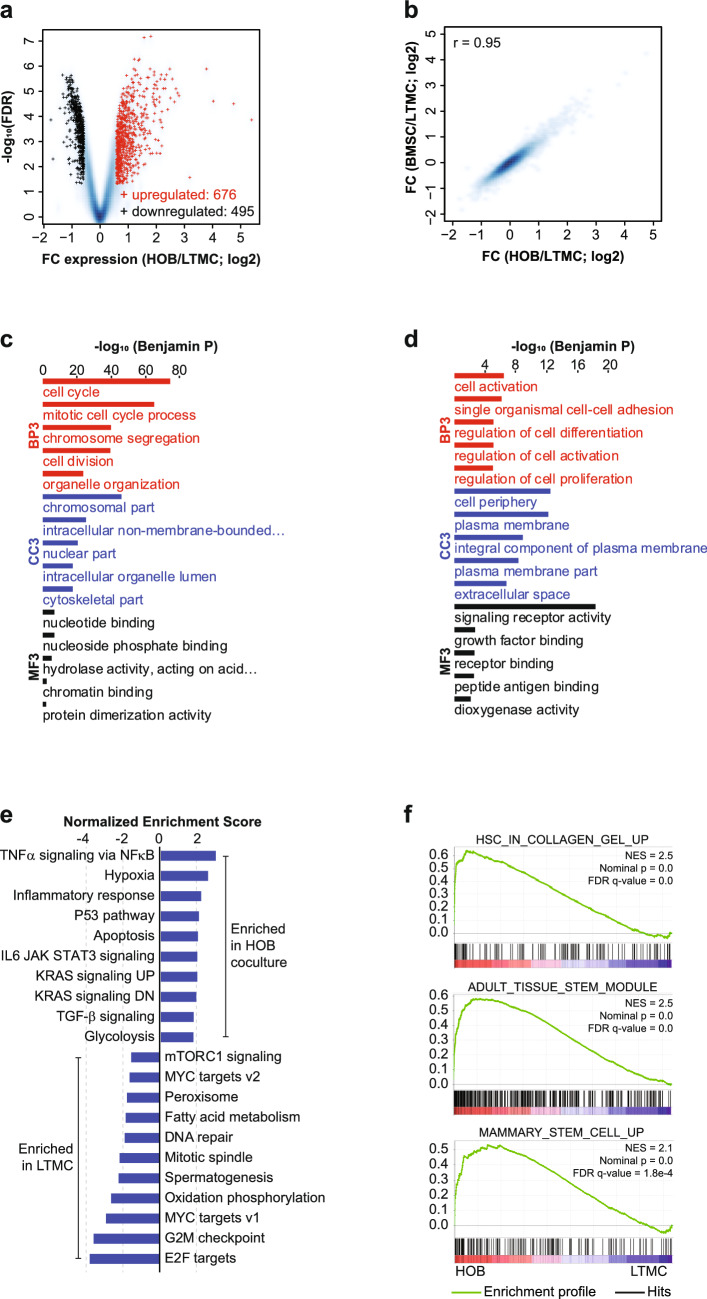
HOB co-culture up-regulated various environment-sensing signaling pathways and down-regulated cell cycle genes. (**a**) Volcano plot for expressed genes between control cells (LTMC) and ALL co-cultured with HOB. Red: up-regulated genes in ALL co-cultured with HOB; Black: down-regulated genes; Blue background: all expressed genes. (**b**) Smoothed scatter plot for the expression changes induced by co-culture of ALL with HOB or with BMSC. *r*: Pearson Coefficient. (**c**) DAVID gene-ontology enrichment analysis on biological processes (BP3), cellular components (CC3), and molecular functions (MF3) for genes down-regulated in ALL co-cultured with HOB. (**d**) DAVID gene-ontology enrichment analysis on biological processes (BP3), cellular components (CC3), and molecular functions (MF3) for genes up-regulated in ALL co-cultured with HOB. (**e**) Bar graphs for the normalized enrichment scores of Gene Set Enrichment Analysis (GSEA) of expressed genes, ranked by the FC of expression (ALL co-cultured/LTMC), against the MSigDB HALLMARK gene sets (FDR q-val < 0.05 and enrichment > 1.5). (**f**) Gene Set Enrichment Analysis (GSEA) of expressed genes, ranked by the FC of expression (ALL co-cultured/LTMC), against the MSigDB gene set “OSWALD HEMATOPOIETIC STEM CELL IN COLLAGEN GEL UP” (top panel), which includes genes up-regulated in hematopoietic stem cells cultured with collagen gel compared to in suspension, gene set “WONG ADULT TISSUE STEM MODULE” (middle panel), which includes genes up-regulated in adult tissue stem cells, and gene set “LIM MAMMARY STEM CELL UP” (bottom panel), which includes genes up-regulated in mammary stem cells.

To characterize the transcriptional program that underlies the quiescent, chemotherapy-resistant phenotype in the co-culture PD cells, we isolated ALL cells from either long-term media culture (LTMC; no microenvironment cues) or from the co-culture with either BMSC or HOB using the method previously described^11^. RNA-Seq analysis was completed on these two ALL cell groups. Analysis of differential gene expression between the LTMC and PD cells co-cultured with HOB resulted in 676 genes up-regulated in the PD cells and 495 genes down-regulated (Fig. 2a). We also completed RNA-Seq using ALL cells cultured with BMSC and found that culturing with HOB or BMSC led to very similar patterns of changes in gene expression (Fig. 2b). We then performed DAVID gene-ontology enrichment analysis for biological processes (BP3), cellular components (CC3), and molecular functions (MF3) for the genes that were down-regulated (Fig. 2c) or up-regulated (Fig. 2d) in the PD cells co-cultured with HOB vs the control cells; similar results were obtained for PD cells co-cultured with BMSCs (Supplementary Fig. S1a,b). The down-regulated genes were involved in cell division and the cell cycle, while the up-regulated genes were enriched in cell adhesion and regulation of cell differentiation. These data are consistent with other publications that have also reported an increase in cellular adhesion makers in models in which leukemic cells have been cultured with non-malignant adherent cells^18–20^. We then conducted a Gene Set Enrichment Analysis (GSEA) to examine the overall expression changes for HALLMARK gene sets defined in MsigDB (FDR q-val < 0.05 and enrichment > 1.5). When compared to the control cells, the PD cells exhibited remarkable enrichments in genes sets for a variety of signaling pathways including TGF-β signaling, hypoxia, and glycolysis (Fig. 2e). The glycolysis signaling pathway is of particular interest, as we have shown that the PD cells are more glycolytically active using a glycolysis stress test and the Seahorse Bioanalyzer, and that they have increased expression of both hexokinase I and II^13^. Additional GSEA revealed remarkable enrichment in the PD cells for gene sets related to cell adhesion and stemness (Fig. 2f). The stemness gene sets included genes expressed by stem cells in adult tissue (ADULT_TISSUE_STEM_MODULE) and genes up-regulated in mammary stem cells (MAMMARY_STEM_CELL_UP). Further transcriptional regulator analysis using LISA^21^ revealed that the down-regulated genes were enriched in the binding of E2F transcription factors and FOXM1, both of which are known regulators of cell proliferation (Supplementary Table S1), while the up-regulated genes were preferentially targeted by ERG, NR3C1, and BHLHE40 (Supplementary Table S2).

**Figure 3:**
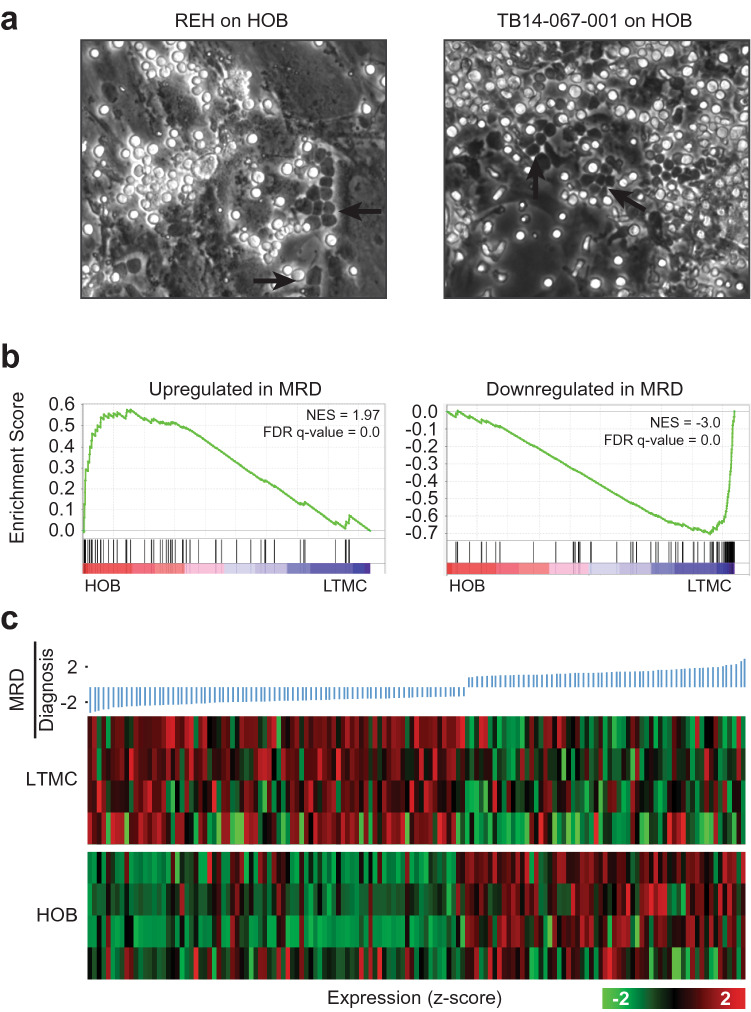
HOB co-culture up-regulated signature genes associated with minimal residual disease of ALL. (**a**) Bright-field microscopy images showing REH co-cultured on HOB (left) and ALL primary patient cells grown on HOB. Arrow heads indicate ALL cell buried under HOB and therefore are referred to as phase-dim (PD). (**b**) Gene Set Enrichment Analysis (GSEA) of expressed genes, ranked by the FC of expression (ALL co-cultured/LTMC), against genes that are up-regulated (left panel) or down-regulated (right panel) in ALL cells corresponding to MRD as compared to ALL cells collected at time of diagnosis and before disease treatment in patients. (**c**) Heat map visualization of expression values in ALL co-cultured with HOB and in suspension (LTMC) for genes differentially expressed between “MRD” and “diagnosis”, sorted by fold-change.

The current RNA-Seq analyses, and our previous data characterizing the co-culture and, specifically, the PD population, motivated us to validate the clinical relevance of our in vitro co-culture model to determine how well it represents cells isolated from B-cell ALL patients, as compared to the traditional cells grown in media alone. We first showed that primary ALL cells from patients could create the same three sub-populations (S, PB, and PD) when co-cultured with HOB (Fig. 3a). To further characterize our co-culture model, we compared our RNA-Seq data from PD cells, isolated from a co-culture of REH B-ALL leukemic cells and HOB, to a public expression data set for MRD^17^. In the paper by Ebinger et al.^17^, they described the gene expression profiles of cells they defined as “having properties of long-term dormancy, treatment resistance, and stemness,” using a xenograft model, which they then used for a comparison to ALL cells isolated from patients having minimal residual disease (MRD). In the Ebinger data set, the authors defined a gene signature for MRD by comparing the gene expression profiles from cells taken at diagnosis compared to gene expression profiles from MRD cells isolated from patients 30 days following the end of treatment. We then applied the GSEA to examine expression changes of the MRD signature genes as defined by Ebinger et al. in our PD cells as compared to control cells (Fig. 3b). We found that the genes up-regulated in the patient derived MRD cells were enriched in the PD cells, while the genes down-regulated in the patient derived MRD cells were enriched in the control cells, demonstrating that the co-culture induced a transcriptional reprogramming towards MRD. We then further visualized the gene expression in our PD cells and the control cells for the signature genes in MRD cells using a heat map. Consistently, we found that genes down-regulated in MRD were expressed at a lower level in PD cells, while those up-regulated in MRD exhibited higher expression in PD cells (Fig. 3c). Similar to the PD cells from a co-culture with HOB, we obtained consistent results for the PD cells obtained from a co-culture with BMSCs (Supplementary Fig. S1c). Collectively, these data suggest that the PD population isolated from our in vitro co-culture model mimics relapsed disease/MRD in patients, and that this in vitro model is clinically relevant (Fig. 3c).

To explore genes and pathways shared between the PD cells and the MRD cells, we identified genes that exhibited consistent expression changes in PD cells vs LTMC cells, and in MRD cells vs the cells collected at diagnosis^17^. We observed a substantial overlap between PD cells and MRD cells for both up-regulated genes and down-regulated genes (Supplementary Fig. S2a). KEGG pathway enrichment analysis for the shared genes is challenging because of the small number of genes. Nevertheless, we observed a shared up-regulation in 15 genes, and shared a down-regulation in 34 genes, many of which involved regulation of the cell cycle (Supplementary Fig. S2a). To further test the hypothesis that PD cells and MRD share common genes and signaling pathways, we focused on expressed genes that are among the top 25% up-regulated in terms of fold-change in PD cells (as compared to LTMC cells), and are also the top 25% up-regulated in MRD cells (as compared to cells at diagnosis). Our analysis on these genes revealed enrichments in KEGG pathways such as cell adhesion molecule (CAMs) and cytokine–cytokine receptor interactions (Supplementary Fig. S2b). Similarly, the top 25% of down-regulated genes in both the PD cells and the MRD cells are enriched in KEGG pathways related to DNA replication and the cell cycle (Supplementary Fig. S2c). To validate the RNA-Seq data showing genes up-regulated in both MRD and PD, we selected a panel of genes and completed qPCR. The genes chosen were *CEBPB*, *SGK1*, *SIGLEC15*, *MVP*, and *ITGB2*, and the validation was completed in the original REH cell line used for the RNA-Seq analysis, as well as two additional ALL cell lines (SUP-B15 and TOM-1) (Supplementary Fig. S3). These genes were selected because they were identified as signature genes for MRD, and there was an overlap in expression with our PD cells. We determined that *CEBPB* and *SGK1* had increased expression in PD cells compared to control cells in the REH, SUP-B15, and TOM1 cell lines. REH and TOM1 PD cells had increased expression of *SIGLEC15*, while SUP-B15 PD cells had increased expression of *MVP*, compared to control cells. The REH and TOM1 PB cells had increased expression of *CEBPB*, *SGK1*, and *SIGLEC15*, while the SUP-B15 PB cells had increased expression of *CEBPB* and *MVP*. C/EBP-β is one of the most up-regulated transcription factors associated with chemotherapy resistance in a model of AML and in a study of multi-drug resistance^22,23^. SGK1 was reported to be up-regulated in both AML, CML, and B-cell lymphoma, and has been shown to play a role in both chemotherapy resistance and radio-resistance due to its structural similarities to AKT^24,25^. SGK1 has also been shown to have roles in regulating autophagy and cell metabolism, both of which are of interest to our laboratory^25^. Siglec-15 is one of many Siglec receptors that interact with sialic acids on the surface of cells, and they are mainly involved in regulation of immune checkpoints^26^. The role of Siglec receptors in cancer is an emerging field of research, but studies have shown that cancer cells are able to utilize these receptors to help suppress and evade the immune system, contributing to cancer immunity and disease progression^26,27^. MVP has also been correlated with chemotherapy resistance, with increased MVP mRNA and protein expression associated with multidrug resistance, and ultimately treatment failure in ALL^28^. It is thought that MVP, along with other ABC transporters and pumps on cell membranes, can either shuttle drugs away from cells or detoxify chemical compounds, thus protecting the cells and allowing tumor progression^28^. Finally, while genes related to cellular adhesion emerged as one of the top gene sets up-regulated in PD cells in our RNA-Seq data, we were unable to detect increased expression of *ITGB2*, a cellular adhesion marker described as a signature gene in MRD that overlapped with our PD cells^17^. *ITGB2* had an overall lower expression compared to the other genes that overlapped in PD cells and MRD, and the varied sensitivities of RNA-Seq and qPCR may have made changes in expression more difficult to detect.

**Figure 4:**
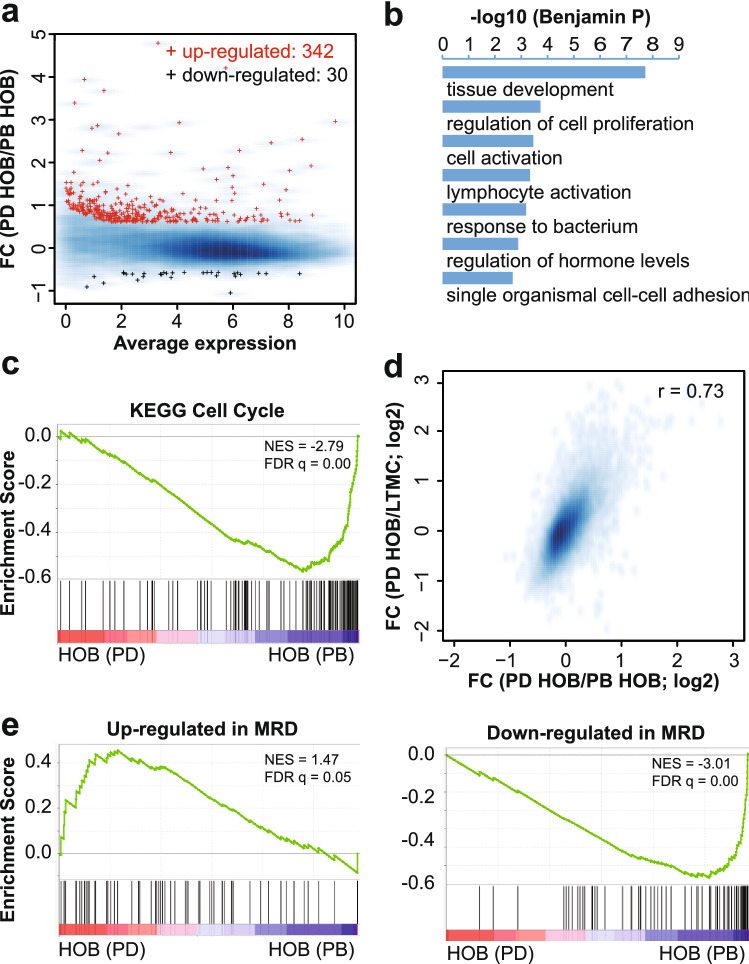
The PD cells are distinctive from the PB cells and are related to MRD. (**a**) MA plot for the average expression and expression fold-changes between PD and PB cells co-cultured with HOB. Red: genes up-regulated from PB to PD; Black: genes down-regulated; blue background: all expressed genes. (**b**) Gene ontology enrichment analysis for genes up-regulated from PB to PD on biological processes (BP3). (**c**) Gene Set Enrichment Analysis (GSEA) of expressed genes, ranked by the FC of expression (PD/PB), against KEGG gene set “Cell cycle”. (**d**) Smoothed scatter plot for the expression changes of PD vs PB (x-axis) and PD vs LTMC (y-axis). *r*: Pearson Coefficient. (**e**) Gene Set Enrichment Analysis (GSEA) of expressed genes, ranked by the FC of expression (PD/PB), against genes that are up-regulated (left panel) or down-regulated (right panel) in ALL cells corresponding to MRD vs control ALL cells collected at time of diagnosis.

Following the analyses of PD cells to control cells, we then compared the transcriptomes of the PD cells and the PB cells to examine their relative connections to the MRD cells. We identified 342 genes up-regulated in the PD cells compared to the PB cells (Fig. 4a). Gene ontology enrichment analysis revealed that these genes are enriched in functions related to biological processes similar to what we have observed for those up-regulated in PD cells compared to the control cells (Figs. 2d and 4b). Gene set enrichment analysis revealed a systematic decrease in the expression levels for genes related to cell cycle (Fig. 4c), consistent with the observations made for the genes down-regulated from LTMC to PD (Fig. 2c). The changes of expression from the PB cells to the PD cells are positively correlated with, and are at a scale comparable to, the changes of expression from the LTMC cells to the PD cells (Fig. 4d). Remarkably, the genes up-regulated in the patient derived MRD cells were preferentially expressed in the PD cells, while the genes down-regulated in the patient derived MRD cells were preferentially expressed in the PB cells (Fig. 4e). While RNA-Seq analyses between the PB and PD cells showed differing patterns of gene expression, our qPCR validation showed that, for the genes assayed, the PB and PD cells had similar trends in expression (Supplementary Fig. S3). Despite looking similar in gene validation experiments by qPCR, we have shown that the PB and PD cells do exhibit functional differences in assays, including those evaluating cell proliferation and sensitivity to chemotherapeutic agents^13,29,30^.

**Figure 5:**
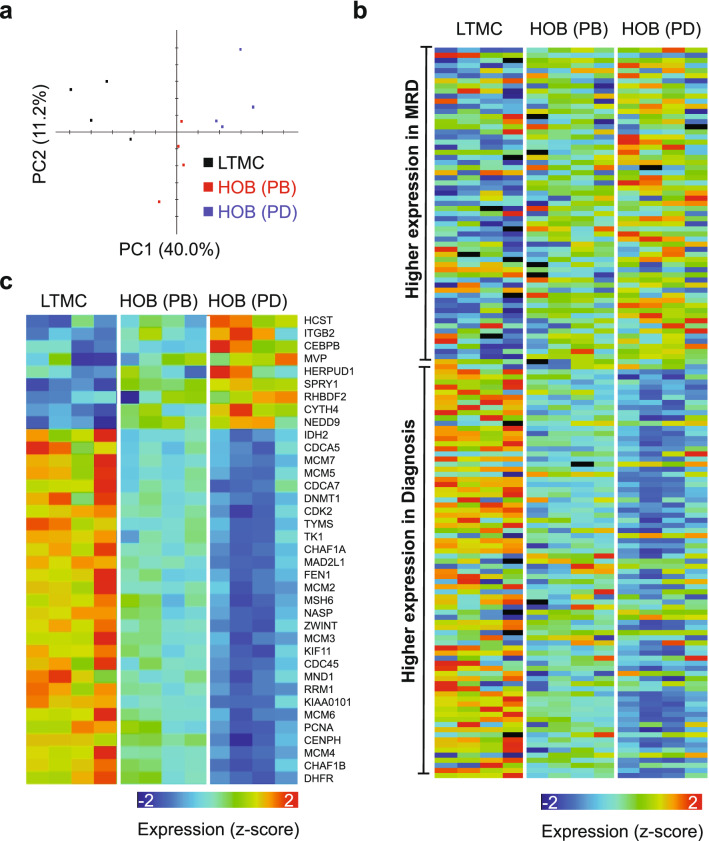
Expression of MRD signature genes in LTMC, PB, and PD cells. (**a**) PCA analysis based the expression of all genes as measured by RNA-Seq analysis for LTMC, PB, and PD cells. (**b**) Heat map visualization of expression values in ALL co-cultured with HOB (PB and PD cells) and in suspension (LTMC) for genes differentially expressed between “MRD” and “diagnosis”, sorted by fold-change. (**c**) Similar to panel b but for genes that are differentially expressed between PD and PB cells.

Finally, to be as thorough as possible, we compared the gene profiles of all 3 in vitro cell populations (LTMC, PB, and PD). A principal component analysis of the three samples based on expression of all genes revealed that PD separates from PB and LTMC (Fig. 5a). It also revealed that the transcription landscape of PB cells could correspond to an intermediate status between LTMC and PD cells (Fig. 5a). We then further compared the expression of MRD signature genes among the three types of cells. The analysis revealed that genes up-regulated in MRD, when compared to disease at diagnosis, were generally expressed at a higher level in PD cells compared to PB or LTMC cells (Fig. 5b). Consistently, genes down-regulated in MRD, when compared to disease at diagnosis, were generally expressed at a lower level in PD cells than LTMC or PB cells (Fig. 5b). From the MRD signature genes, we then highlighted a subset that were differentially expressed between PD and PB and compared their expression across the three cell populations. This analysis demonstrated that the PB cell population is an intermediate between LTMC and PD cells (Fig. 5c). Therefore, the PD cells constitute a unique cell population with a gene expression profile that is the most closely related to the gene profile described for MRD in ALL.

## Discussion

The development of novel chemotherapeutics for B-cell ALL have been hampered by the lack of in vitro model systems that can recapitulate the cellular signals seen in human patients. Increasing evidence supports the bone marrow niche as a critical contributing factor to the tumor microenvironment supporting MRD^13,31^. While there has been significant progress in the treatment of B-cell ALL, with most pediatric and adult patients achieving complete remission following treatment with the current standard-of-care therapeutic regimens, there is still a subset of patients who will experience a disease relapse, due to MRD. The presence of MRD following the completion of the treatment regimen is one of the most important prognostic factors for determining which patients will likely relapse. Often the newly emerging/relapsed disease has a chemotherapy-resistant phenotype, reducing the possibility of a complete remission for these patients^7^.

Identification of a transcription signature of ALL MRD would facilitate the development and evaluation of therapeutic agents specifically targeting this drug-resistant population contributing to relapse. This unmet need underpins the necessity of a reproducible model that is both available for efficient evaluation and also clinically relevant. We have developed an in vitro co-culture model to study MRD, using a combination of B-cell ALL cells and human bone marrow microenvironment specific cells that segregates ALL cells into sub-populations, with the phase dim (PD) cell population exhibiting a quiescent, chemotherapy-resistant phenotype. Using RNA-Seq analyses, we demonstrated that the PD ALL cell population was characterized by a transcription program similar to that of MRD cells isolated from patients. These results provided validation that our in vitro co-culture model generates a cell population (PD) that closely recapitulates many of the characteristics found in MRD. Since the bone marrow environment has been shown to contribute to support of ALL cell viability even during aggressive therapy, and protects residual cells that are poised to contribute to relapse of disease following cessation of therapy, the inclusion of the adherent cells (HOB or BMSC) is required for recapitulating the transcription profile observed in patients; in contrast to cells which are normally cultured in media alone. The characterization of in vitro models that can be used to study the molecular pathways involved in tumor cell resistance and quiescence have significant utility. Critical evaluation of the relevance of these models is enabled by patient-derived data from other laboratories that make essential comparisons possible, and will allow for identification of novel therapeutic strategies to treat MRD.

## Methods

### Cell culture

REH (ATCC #CRL-8286), TOM-1 (DSMZ ACC#578), and SUPB15 (ATCC #CRL-1929) were purchased and maintained in RPMI 1640 supplemented with 10% FBS and 1 × streptomycin/penicillin antibiotics. Human osteoblasts (HOB) were purchased from PromoCell (Cat No: C-12720, Heidelberg, Germany) and cultured according to the vendor’s recommendations. De-identified primary BMSC and B-cell ALL samples were provided by the WVU Cancer Institute Biospecimen Processing Core and the WVU Department of Pathology Tissue Bank. ALL cell lines were authenticated by short tandem repeat (STR) analysis (University of Arizona Genetics Core, Tucson, AZ) and maintained in 6% CO_2_ in normoxia at 37 °C.

### Co-culture model

Co-culture conditions were followed as previously described^11^. Briefly, 1 million ALL cells were seeded on an 85% confluent BMSC or HOB layer and maintained in 5% O_2_. The co-culture was fed every 4 days. On the 12th day in culture, the ALL cells were isolated for further processing. The leukemic cell population that was in suspension and not interacting with the stromal cells was collected and designated as phase bright cells (PB). The leukemic cells that were buried under the BMSC or HOB were separated by size exclusion with Sephadex G-10 after vigorous washing to remove all leukemic cells adhered to the top of the BMSC. Buried leukemic cells were designated phase dim cells (PD). To validate the RNA-Seq analysis, REH, SUP-B15, and TOM-1 leukemic cells were grown in media alone or co-culture, and separated as described above (n = 3 for each cell line). RNA was isolated from the leukemic cell pellets from the LTMC and PD groups, and RT-PCR completed using primers for *CEBPB*, *SGK1*, *SIGLEC15*, *MVP*, *ITGB2*, and *RPL13A* (all from Real-Time Primers).

### RNA-Seq analysis

The RNA-Seq library preparation were previously described^32^. Briefly, RNA was isolated from the purified ALL cells with a RNeasy Plus Mini Kit (Qiagen, Hilden, Germany), and samples were sent to the West Virginia University Genomics Core for library preparation using polyadenylation selection with the KAPA Stranded mRNA-Seq Kit. RNA-Seq libraries were sequenced using a HiSeq 1500 system (50 bp paired-end) (Illumina, San Diego, CA, USA). To complete the RNA-Seq data analyses, we followed the methods described in our previous publications^32,33^. Briefly, the subread package^34^ to map the pair-end RNA-Seq reads to the human genome (hg38), and the *featureCounts* function within the Rsubread R package^35^ to summarize the number of reads for genes annotated by RefSeq were utilized. Gene expression level was quantified by Reads Per Kilobase of transcript, per Million mapped reads (RPKM)^36^. Downstream analyses considered only protein-coding genes that expressed in at least one condition (RPKM > 2) and excluded histone genes. Differentially expressed genes were predicted using EdgeR 3 under a fold-change (FC) > 1.5 and a false discovery rate (FDR) < 0.05. The online DAVID Bioinformatics resource was used for gene-ontology enrichment analysis using the differentially expressed genes as a foreground and all expressed genes as a background. Predictions of transcription regulators for differentially expressed genes were done with LISA^21^. GSEA 4.0.2 was utilized for gene set enrichment analysis taking genes sorted by FC of expression as input, and calculated the enrichment for interested gene sets extracted from MSigDB^37^. Additional gene sets for GSEA analysis included genes up-regulated or down-regulated in minimal residual cells as compared to cells collected at time of diagnosis, obtained from “Table S8” published by another laboratory^17^. MeV^38^ enabled visualization of gene expression in a heat map.

## Supplementary Information

Supplementary Legends.

Supplementary Figure S1.

Supplementary Figure S2.

Supplementary Figure S3.

Supplementary Table S1.

Supplementary Table S2.

## Data Availability

RNA-Seq data were deposited to GEO with accession GSE148520.
