# Supplementary material for: Co-culture model of B-cell acute lymphoblastic leukemia recapitulates a transcription signature of chemotherapy-refractory minimal residual disease
Source: Sci Rep. 2021 Aug 4;11:15840. doi: 10.1038/s41598-021-95039-x (PMC8339057; doi:10.1038/s41598-021-95039-x)
Supplement: Supplementary file 1 — Supplementary Legends. [file 41598_2021_95039_MOESM1_ESM.docx]

**Supplementary Data:**

**Co-culture model of B-cell acute lymphoblastic leukemia recapitulates a transcription signature of chemotherapy-refractory minimal residual disease**

Stephanie L. Rellick, Gangqing Hu, Debbie Piktel, Karen H. Martin, Werner J. Geldenhuys, Rajesh R. Nair, and Laura F. Gibson

**Supplementary Figure Legends**

**Supplementary Figure S1: Co-culture with BMSCs induced expression changes similar to the coculture with HOBs.**

1. Bar graphs for the normalized enrichment scores of GSEA analysis of expressed genes, ranked by the FC of expression (ALL co-cultured with BMSCs/LTMC), against the MSigDB HALLMARK gene sets (FDR q-val < 0.05 and enrichment > 1.5).
2. GSEA analysis of expressed genes, ranked by the FC of expression (ALL co-cultured with BMSCs/LTMC), against the MSigDB gene set “OSWALD HEMATOPOIETIC STEM CELL IN COLLAGEN GEL UP”, which includes genes up-regulated in hematopoietic stem cells cultured with collagen gel compared to in suspension, and gene set “WONG ADULT TISSUE STEM MODULE” (middle panel), which includes genes up-regulated in adult tissue stem cells.
3. GSEA analysis of expressed genes, ranked by the FC of expression (ALL co-cultured with BMSCs/LTMC), against genes that are up-regulated or down-regulated in ALL cells corresponding to MRD as compared to ALL cells collected at time of diagnosis and before disease treatment in patients.

**Supplementary Figure S2: Shared pathways between PD cells and MRD cells.**

1. Left panel: Venn diagram for genes up-regulated in PD cells vs LTMC cells and in MRD cells vs the control cells collected at diagnosis; right panel: Venn diagram for genes down-regulated in PD cells and in MRD cells.
2. KEGG enrichment analysis for expressed genes that are among the top 25% up-regulated in terms of fold-change in PD cells vs LTMC cells and also the top 25% up-regulated in MRD cells vs the control cells.
3. KEGG enrichment analysis for expressed genes that are among the top 25% down-regulated in terms of fold-change in PD cells vs LTMC cells and also the top 25% down-regulated in MRD

cells vs the control cells.

**Supplementary Figure S3: Validation of transcription signatures identified from RNA-Seq in PB and PD cells using three ALL cell lines**.

REH, SUP-B15, and TOM-1 leukemic cell lines were grown in media (LTMC) or in co-culture with human osteoblasts (HOB) for 10 days. Both media and co-culture cells were then subjected to separation using G-10 Sephadex. RNA was extracted from the isolated leukemic cells and qPCR completed for *CEBPB*, *SGK1*, *SIGLEC15*, *MVP*, and *ITGB2*. *RPL13A* was used for normalization. The average fold change of each gene is graphed with standard error (SEM), with the black line indicating LTMC, which was used for comparison.

**Supplementary Table S1: Potential regulators (top 20) for genes down-regulated in PD as compared to LTMC**

**Supplementary Table S2: Potential regulators (top 20) for genes up-regulated in PD as compared to LTMC**
