# Supplementary figures and images for: Co-culture model of B-cell acute lymphoblastic leukemia recapitulates a transcription signature of chemotherapy-refractory minimal residual disease
Source: Sci Rep. 2021 Aug 4;11:15840. doi: 10.1038/s41598-021-95039-x (PMC8339057; doi:10.1038/s41598-021-95039-x)

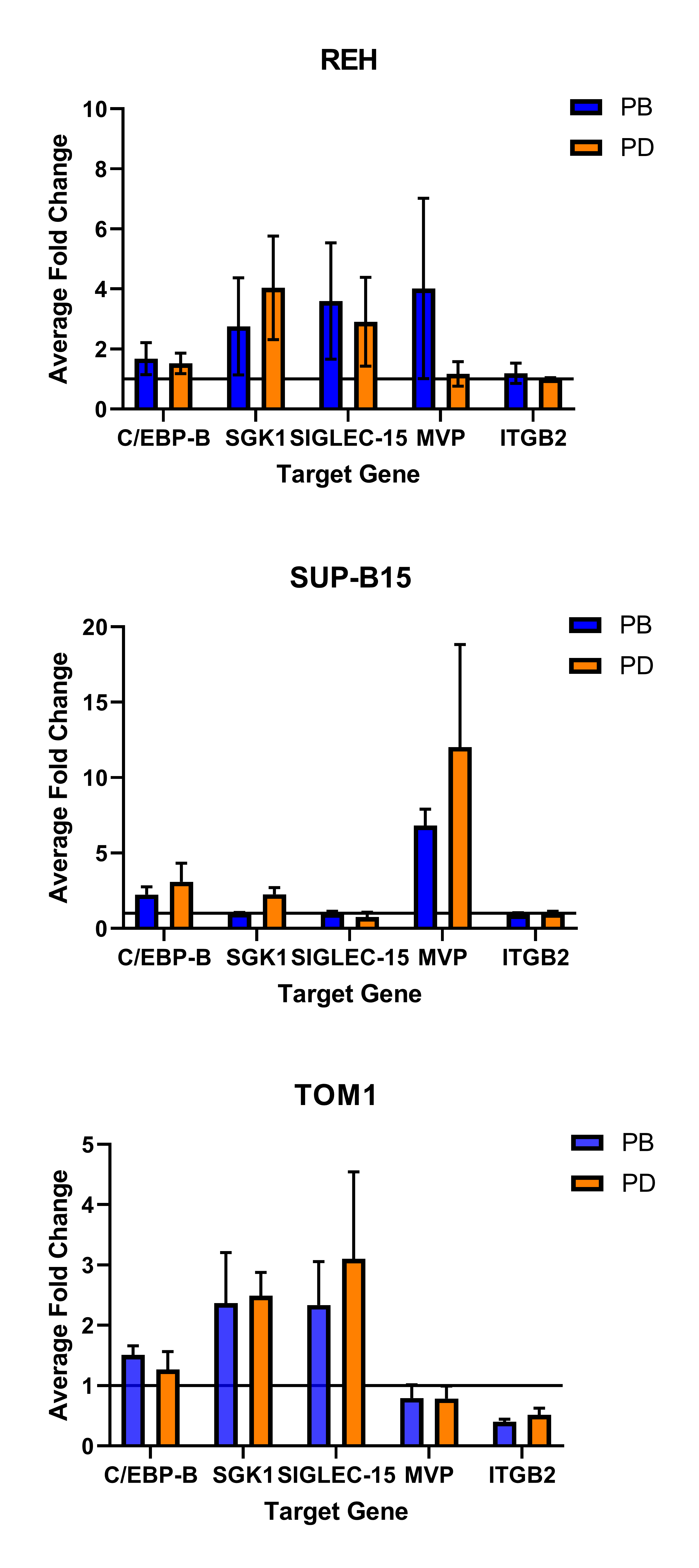

Supplement: Supplementary file 4 — Supplementary Figure S3. [file 41598_2021_95039_MOESM4_ESM.tif]
