# Supplementary material for: Co-culture model of B-cell acute lymphoblastic leukemia recapitulates a transcription signature of chemotherapy-refractory minimal residual disease
Source: Sci Rep. 2021 Aug 4;11:15840. doi: 10.1038/s41598-021-95039-x (PMC8339057; doi:10.1038/s41598-021-95039-x)
Supplement: Supplementary file 5 — Supplementary Table S1. [file 41598_2021_95039_MOESM5_ESM.docx]

| Gene symbol | -LOG10(p-value) from LISA |
| --- | --- |
| E2F4 | 137.4 |
| E2F1 | 115.8 |
| RBL2 | 97 |
| TFDP1 | 93.6 |
| RNF2 | 62.7 |
| FOXM1 | 60.7 |
| ING2 | 58.9 |
| HDAC1 | 58.7 |
| RB1 | 57.8 |
| FOXP1 | 57.6 |
| HDAC2 | 55.8 |
| HBP1 | 54.9 |
| TAF1 | 54.8 |
| SAP30 | 53.9 |
| NELFA | 52.7 |
| GTF2B | 52.3 |
| XRN2 | 49.8 |
| SIN3A | 49.1 |
| PHF8 | 48.5 |
| CDK9 | 48.1 |

**Supplementary Table S1: Potential regulators (top 20) for genes down-regulated in PD as compared to LTMC**
