# Supplementary material for: Co-culture model of B-cell acute lymphoblastic leukemia recapitulates a transcription signature of chemotherapy-refractory minimal residual disease
Source: Sci Rep. 2021 Aug 4;11:15840. doi: 10.1038/s41598-021-95039-x (PMC8339057; doi:10.1038/s41598-021-95039-x)
Supplement: Supplementary file 6 — Supplementary Table S2. [file 41598_2021_95039_MOESM6_ESM.docx]

**Supplementary Table S2: Potential regulators (top 20) for genes up-regulated in PD as compared to LTMC**

| Gene symbol | -LOG10(p-value) from LISA |
| --- | --- |
| ERG | 55.4 |
| NR3C1 | 52.7 |
| TCF4 | 50.7 |
| RUNX1 | 47.8 |
| IKZF1 | 46 |
| EGR3 | 45 |
| FLI1 | 42.5 |
| KMT2A | 41.9 |
| TERC | 41.5 |
| KDM2B | 40.5 |
| CTCF | 40 |
| BHLHE40 | 39.7 |
| MYC | 39.1 |
| PML | 39 |
| EGR2 | 38.9 |
| EBF1 | 38.9 |
| MAX | 38.8 |
| MYB | 38.3 |
| MAZ | 38.1 |
| JMJD1C | 38.1 |
